# Integrated Cytological, Physiological, and Transcriptome Analyses Provide Insight into the Albino Phenotype of Chinese Plum (*Prunus salicina*)

**DOI:** 10.3390/ijms241914457

**Published:** 2023-09-22

**Authors:** Weiwei Xie, Dantong Xu, Fangce Chen, Zhengpeng Wang, Jiandong Luo, Yehua He, Qianming Zheng, Chaoyang Liu

**Affiliations:** 1 Key Laboratory of Biology and Germplasm Enhancement of Horticultural Crops in South China, Ministry of Agriculture and Rural Affairs, College of Horticulture, South China Agricultural University, Guangzhou 510642, China; weiweixievivi@foxmail.com (W.X.); d1048516541@163.com (D.X.); cfc0613@stu.scau.edu.cn (F.C.); wangzhengpeng2940@163.com (Z.W.); 19303032781@163.com (J.L.); heyehua@scau.edu.cn (Y.H.); 2 Maoming Branch, Guangdong Laboratory for Lingnan Modern Agriculture, Maoming 525000, China; 3 Institute of Pomology Science, Guizhou Academy of Agricultural Science, Ministry of Agriculture and Rural Affairs Key Laboratory of Crop Genetic Resources and Germplasm Innovation in Karst Region, Guiyang 550006, China

**Keywords:** *Prunus salicina*, plum, albino, chloroplast development, transcriptomes

## Abstract

Albino seedlings that arise during seed reproduction can have a significant impact on plant growth and breeding. In this research, we present the first report of albino occurrences in the seed reproduction process of *Prunus salicina* and describe the cytological, physiological, and transcriptomic changes observed in albino seedlings. The albino seedlings which were observed in several plum cultivars exhibited abnormal chloroplast ultrastructure and perturbed stomatal structure. Compared to normal seedlings, the photosynthetic pigment contents in albino seedlings decreased by more than 90%, accompanied by significant reductions in several chlorophyll fluorescence parameters. Furthermore, substantially changed photosynthetic parameters indicated that the photosynthetic capacity and stomatal function were impaired in albino seedlings. Additionally, the activities of the antioxidant enzyme were drastically altered against the background of higher proline and lower ascorbic acid in leaves of albino seedlings. A total of 4048 differentially expressed genes (DEGs) were identified through transcriptomic sequencing, and the downregulated DEGs in albino seedlings were greatly enriched in the pathways for photosynthetic antenna proteins and flavonoid biosynthesis. *GLK1* and *Ftsz* were identified as candidate genes responsible for the impaired chloroplast development and division in albino seedlings. Additionally, the substantial decline in the expression levels of examined photosystem-related chloroplast genes was validated in albino seedlings. Our findings shed light on the intricate physiological and molecular mechanisms driving albino plum seedling manifestation, which will contribute to improving the reproductive and breeding efforts of plums.

## 1. Introduction

Plums belong to the *Rosaceae* family and the *Prunus* genus. There are over 6000 cultivated varieties of plums distributed worldwide [1]. The most important commercial cultivars of plums are the European plum (*Prunus domestica*) and Chinese plum (*P. salicina*) and their hybrids. The propagation of plums often uses methods like grafting, cutting, seed propagation, and air layering. Among them, seed propagation is a cost-effective way to produce offspring with high genetic diversity, which plays an important role in the new variety breeding of plums [2].

The albino phenotypes are common in the plant kingdom and are frequently observed in seed-propagated seedlings. Some albino seedlings can recover their green color with changes in temperature [3] and proper plant growth and development [4]. However, the majority of them face a fatal outcome due to factors like impaired photosynthesis. A prime example of this is the *Arabidopsis thaliana* albino mutant *pbf2*, which lacks the PSI complex, rendering it unable to conduct photosynthesis and ultimately leading to its demise [5]. The occurrence of natural albino phenotypes has been studied in a few woody plants. For example, in arecanut (*Areca catechu* L.) seedlings, the proportion of seedlings with striped albino leaf was about 0.2%, and these affected seedlings typically died within several months [6]. Similarly, albino coconut seedlings cannot use internal iron, and despite external iron improving chloroplast development, the plants eventually die [7]. In royal poinciana (*Delonix regia*) seedlings, albino seedlings may emerge but only survive for about 3 weeks until the cotyledons became completely senescent [8]. Additionally, albino seedlings have also been observed in several fruit trees such as jackfruit [9], citrus [10], and apple [11].

Existing research has shown that albino seedlings not only exhibit differences in photosynthetic pigment content compared to normal seedlings but also display abnormalities in chloroplast morphology, leaf epidermis, and other aspects. These characteristics often lead to the inability of plants to carry out essential life processes like photosynthesis and respiration, ultimately resulting in death. Changes in chloroplast morphology and number are commonly observed in the leaves of albino seedlings. For example, the loss of function of *CsTIC21* in cucumber seedlings led to chloroplast malformation, with distorted structure and reduced thylakoid accumulation, resulting in albino leaves and eventual death [12]. In poplar, the loss of *PtrDJ1C* resulted in albino seedlings with almost no observable chloroplasts in their leaves [13]. Albino seedlings also exhibit significant changes in the leaf epidermis. Studies have demonstrated that the *dxr* mutant exhibited albino and dwarf phenotypes with abnormal leaf features, including significantly reduced trichomes and impaired stomatal closure [14]. In the albino mutant of *Agave angustifolia*, significant differences in the leaf epidermis compared to green leaves were observed [15,16].

There are several reasons that can cause seedling albino phenotypes, such as mutations in genes involved in the synthesis of plant photosynthetic pigments [17], mutations affecting chloroplast formation and development-related genes [18], incompatibility in nuclear–cytoplasmic gene interactions [19], and environmental factors like temperature [20]. Albino seedlings usually exhibit significant differences from normal plants at the gene expression level. Comprehensive gene expression information can be obtained through high-throughput transcriptome sequencing, which is helpful for deciphering the potential mechanism of the albino formation. For example, transcriptome analyses revealed that the molecular mechanism for albino leaf color formation in wheat albino mutant *mta* was a thoroughly regulated and complicated process involved in chloroplast development, chlorophyll biosynthesis, and photosynthesis [21]. De novo transcriptome analysis revealed that the reduction in chloroplast biosynthesis and division in the *Pasphiopedilum pacific shamrock* albino mutant was possibly due to low expression levels of *ppGLK1* and *ppFtsz* [22]. The combination of transcriptome and GWAS data identified a series of differentially expressed genes involved in porphyrin and chlorophyll metabolism, which might affect the formation of albino propagules in *Kandelia obovata* [23].

Albino phenomena in the *Rosaceae* family have been documented in several reports, with instances attributed to virus infections [24] or observed during tissue culture [25]. Noteworthily, albino seedlings have also been reported in the hybridization breeding program of sweet cherries (*Prunus avium* L.) through seed propagation [26]. Furthermore, during the selfing process of the European plum cultivar ‘Ortenauer’, seedlings with chlorophyll deficiency were also discovered [27]. However, there is currently a lack of research reports on albino phenomena during seed propagation in the Chinese plum. The Chinese plum is one of the most abundant and variable groups of fruit tree species, and many hybrids of this species have been made and used either as cultivars or rootstocks [1]. The efficiency of seed reproduction is one of the important factors that need to be considered in the hybrid breeding program of the Chinese plum. Owing to the albino phenomena which occurred in seed propagation, more progeny seedlings had to be obtained to compensate for the losses caused by albinism, which will significantly increase the burden of breeding work and reduce the efficiency of the crossbreeding and seedling selection. Therefore, this study focuses on albino seedlings that occur during the seed propagation process of the Chinese plum. By investigating their chloroplast and stomatal morphology, physiological indicators, the nuclear transcriptome, and the expression of the photosynthesis-related chloroplast genes, we aim to analyze the reasons for the impaired growth of albino seedlings to provide relevant evidence for studying the mechanisms underlying the formation of albino seedlings during Chinese plum seed propagation. This research will contribute to the broader understanding of albino phenomena in the *Rosaceae* family and potentially aid in improving seed propagation practices and breeding efforts for Chinese plum cultivars.

## 2. Results

### 2.1. Observation of Embryo Germination and Seedling Phenotype Characterization

**Figure 1:**
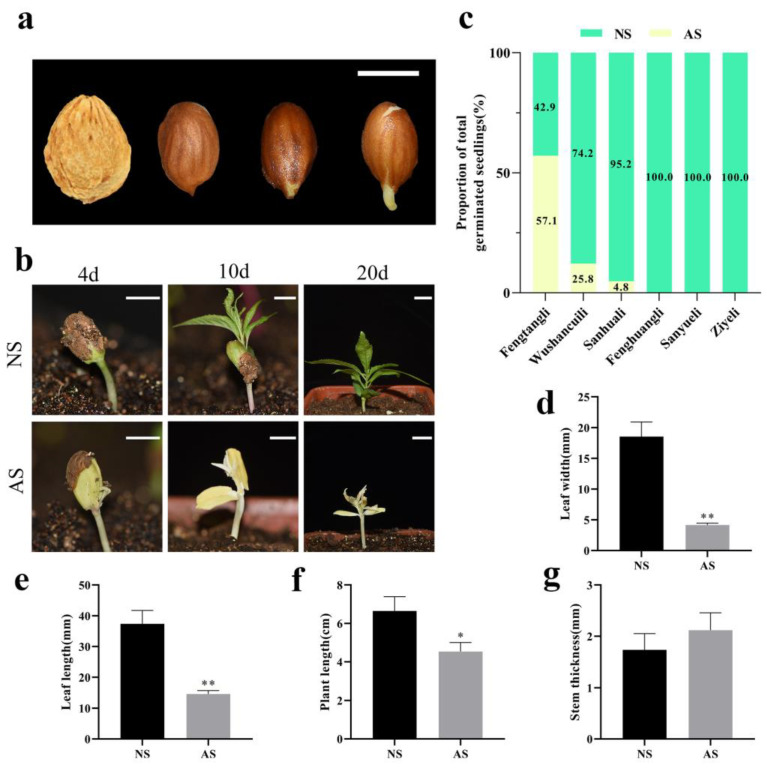
Seed germination under low-temperature treatment and seedling growth of *P. salicina*. (**a**) Embryo root growth process during low-temperature treatment of *P. salicina* seeds. (**b**) Normal seedlings and albino seedlings of *P. salicina* 4, 10, and 20 days after sowing. (**c**) Proportion of total germinated seedlings. (**d**) Leaf width. (**e**) Leaf length. (**f**) Plant length. (**g**) Stem thickness. NS: normal seedlings; AS: albino seedlings; error bars represent mean ± SD (*n* = 3). Scale bar = 1 cm (**a**,**b**). Asterisks indicate the following: * *p* < 0.05; ** *p* < 0.01.

After removing the outer shell, the embryos were obtained and treated in low-temperature conditions, and subsequently, the embryonic roots emerged and elongated gradually (Figure 1a). Once the embryonic roots reached a length of approximately 0.5 cm, the seeds were planted into substratum. After about 4 days, the seedlings emerged from the substratum. At this stage, the cotyledons of the seedlings were tightly closed, and the brown seed coat had not completely shed, making it possible to differentiate between normal and albino seedlings. The cotyledons of albino seedlings appeared yellow. Ten days after sowing, the true leaves of the seedlings unfolded, which appeared green and albino in normal and albino seedlings, respectively. Twenty days after sowing, normal seedlings continued to grow normally, while the leaves of albino seedlings turned brown and gradually withered, exhibiting lethal albino characteristics (Figure 1b). Statistical analysis showed that the proportion of albino seedlings varied greatly among different varieties (Figure 1c). Among them, the variety of ‘Fengtangli’ and ‘Wushancuili’ exhibited a high proportion of albino seedlings (>25%), while no albino seedlings were found among the varieties of ‘Fenghuangli’, ‘Sanyueli’, and ‘Ziyeli’. Morphological measurements were performed 15 days after sowing. The observations revealed that albino seedlings exhibited significantly shorter leaf width (Figure 1d), leaf length (Figure 1e), and plant height (Figure 1f) compared to normal seedlings. However, there was no significant difference in stem thickness between the two groups (Figure 1g). Throughout their survival period, the growth of albino seedlings was much slower than that of normal seedlings.

### 2.2. Measurement of Photosynthetic Pigment Content and Ultrastructural Observation of Chloroplasts in Leaves of Seedlings

**Figure 2:**
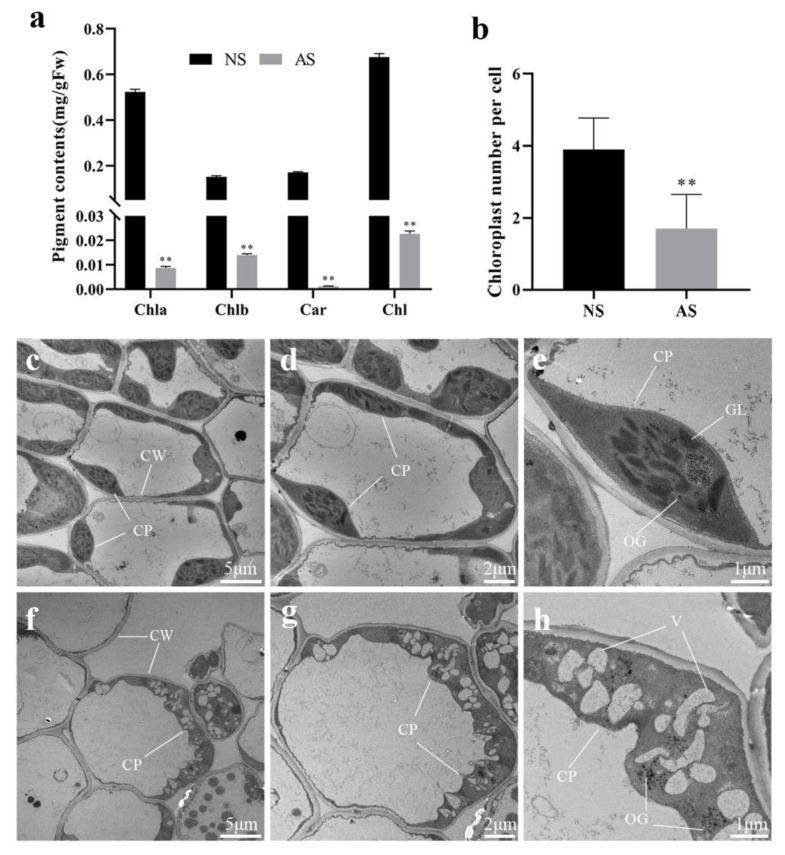
Pigment content and chloroplast ultrastructure in leaves of normal and albino seedlings of *P. salicina*. (**a**) Pigment content of normal and albino seedlings of *P. salicina*. (**b**) The average number of chloroplasts per cell. (**c**–**h**) Chloroplast ultrastructure on normal leaves (**c**–**e**) and albino leaves (**f**–**h**). NS: normal seedlings; AS: albino seedlings; Chla: chlorophyll a; Chlb: chlorophyll b; Car: carotenoid; Chl: chlorophyll a + chlorophyll b; CP: chloroplast; CW: cell wall; V: vacuole; GL: grana lamella; OG: osmiophilic granule; error bars represent mean ± SD (*n* = 3). Asterisks indicate the following: ** *p <* 0.01.

The albino seedling exhibited an overall yellowish-white color. Further analysis revealed that a small amount of chlorophyll still existed in the albino seedlings. However, the levels of chlorophyll and carotenoid in albino seedlings were significantly lower compared to those in normal seedlings (Figure 2a). In albino seedlings, the content of chlorophyll a, chlorophyll b, total chlorophyll, and carotenoid decreased by 98.28%, 90.79%, 96.59%, and 99.41%, respectively, compared to normal seedlings. The significant decrease in the photosynthetic pigment content could seriously affect normal photosynthesis in albino seedlings.

The presence of albino traits is frequently accompanied by alterations in chloroplast structure. Therefore, we investigated chloroplasts using transmission electron microscopy in both normal and albino seedlings. Notably, the leaves of albino seedlings exhibited a significantly lower average number of chloroplasts per cell compared to normal green leaves (Figure 2b). In mesophyll cells of normal seedlings, the chloroplasts appeared intact with well-defined thylakoid membranes and stromal lamellar structures, showing only a few osmiophilic granules (Figure 2c–e). Conversely, chloroplasts in the leaves of albino seedlings showed ruptured thylakoid membranes and fragmented lamellar structures. Additionally, numerous vesicles and osmiophilic granules were observed (Figure 2f–h). These characteristics implied a significant impairment of photosynthetic function in the leaves of albino seedlings.

### 2.3. Chlorophyll Fluorescence and Photosynthetic Parameters in Leaves of Seedlings

Through the analysis of chlorophyll fluorescence parameters in both types of seedlings, it is evident that albino seedlings have significantly lower values for the initial fluorescence (F_0_), maximum fluorescence (Fm), non-photochemical quenching coefficient (NPQ), photochemical quenching coefficient (qP), and maximum photochemical efficiency of PSII (Fv/Fm) compared to normal seedlings (Table 1), which were reduced by 51.73%, 84.34%, 79.73%, 56.90%, and 25%, respectively. The net photosynthetic rate (Pn) of leaves from albino seedlings exhibited a significant reduction of 191.58% compared to that from normal seedlings. In contrast, stomatal conductance (Gs) and intercellular CO_2_ concentration (Ci) showed marked increases of 340.25%, 28.27%, and 149.29%, respectively, in albino seedlings compared to normal seedlings. These observations strongly suggest that the leaves of albino seedlings are impaired in their ability to carry out photosynthesis effectively.

### 2.4. Stomatal Development in Leaves of Seedlings

**Figure 3:**
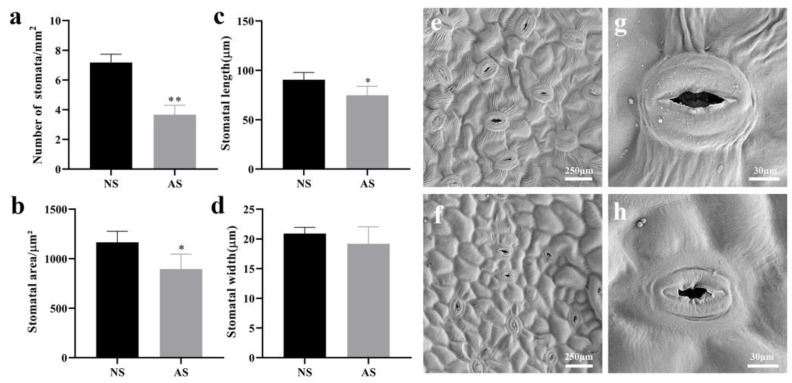
Leaf surface characteristics in normal and albino seedlings of *P. salicina.* (**a**) Number of stomata. (**b**) Stomatal area. (**c**) Stomatal length. (**d**) Stomatal width. (**e**–**h**) Micrographs obtained using scanning electron microscopy of leaf surfaces for both normal and albino seedlings. NS: normal seedlings; AS: albino seedlings. Scale bars = 250 μm (**e**,**f**), 30 μm (**g**,**h**). Error bars represent mean ± SD (*n* = 3). Asterisks indicate the following: * *p <* 0.05; ** *p <* 0.01.

Scanning electron microscopy observation of the lower epidermis of seedling leaves revealed significant differences between normal and albino seedlings. In albino seedlings, a significantly lower number (Figure 3a), smaller areas (Figure 3b), and shorter lengths of stomata (Figure 3c) were observed, compared to those in the lower epidermis of normal seedling leaves. Additionally, notable differences in stomatal width between the two groups were not found (Figure 3d). In the lower epidermis of normal seedling leaves, there were numerous wrinkles around the guard cells due to the opening and closing of the stomata (Figure 3e). In contrast, the lower epidermis of albino seedling leaves appeared smoother around the stomata (Figure 3f). Moreover, distinct differences in the morphology of the stomata were observed between the two groups. The stomatal apparatus in albino seedlings lacked well-defined guard cells and appeared collapsed, whereas the guard cells in the lower epidermis of normal seedlings were more pronounced (Figure 3g,h). Meanwhile, the transpiration rate (E) in albino seedlings increased by 149.29% compared to normal seedlings (Table 1). This phenomenon may indicate that the gas exchange in albino seedlings during growth cannot be properly regulated.

### 2.5. Antioxidant Enzyme Activity and Levels of Glutathione, Ascorbic Acid, and Proline in Leaves of Seedlings

**Figure 4:**
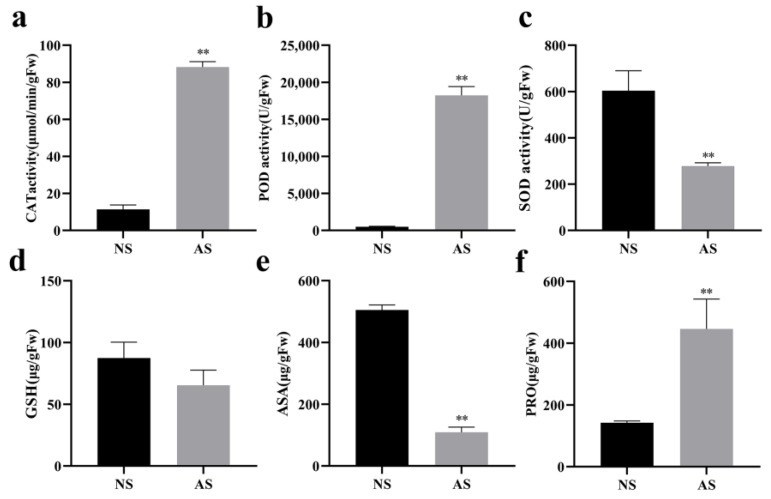
Enzyme activity and content of glutathione, ascorbic acid, and proline in leaves of normal and albino seedlings of *P. salicina*. (**a**) Catalase (CAT) enzyme activity. (**b**) Peroxidase (POD) enzyme activity. (**c**) Superoxide dismutase (SOD) enzyme activity. (**d**) Content of glutathione (GSH). (**e**) Content of ascorbic acid (ASA). (**f**) Content of proline (PRO). NS: normal seedlings; AS: albino seedlings. Error bars represent mean ± SD (*n* = 3). Asterisks indicate the following: ** *p* > 0.01.

The activities of POD (peroxidase), SOD (superoxide dismutase), and CAT (catalase) enzymes, as well as the contents of GSH (glutathione), ASA (ascorbic acid), and PRO (proline), were determined in leaves of albino and normal seedlings. The results showed that the activities of CAT and POD enzymes (Figure 4a,b) in albino seedlings were significantly higher than those in normal seedlings, while the activity of SOD was lower (Figure 4c). There was no significant difference in GSH content between the two types of seedlings (Figure 4d). The ASA content in albino seedlings was significantly lower than in normal seedlings (Figure 4e), while the PRO content was significantly higher than in normal seedlings (Figure 4f).

### 2.6. Transcriptomic Alterations in Leaves of Seedlings

To reveal the molecular events occurring in the albino seedlings, transcriptome sequencing and DEG analysis were performed using normal and albino seedling leaves of Chinese plum. A cDNA library was constructed for each sample, and a total of 40.64 G raw data with high quality was generated (Table S1). The average GC contents were approximately 46.39% and 45.67%, and the average Q30 values were 92.81% and 92.24% in the normal and albino seedling, respectively. The sequencing raw data were deposited into the NCBI Sequence Read Archive (SRA) with the accession number PRJNA1005015.

**Figure 5:**
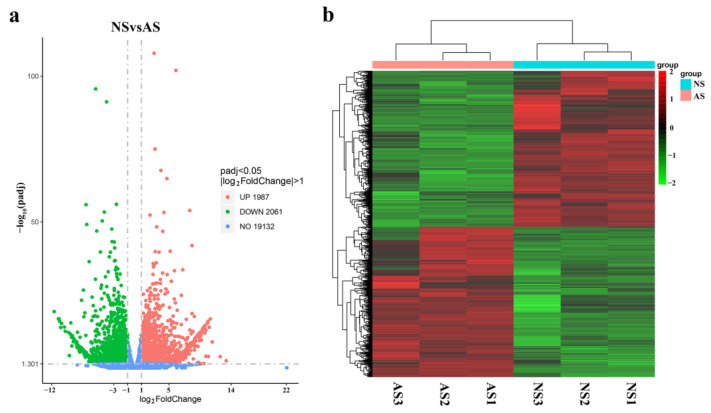
Transcriptomic comparison between the leaves of normal and albino seedlings of *P. salicina.* (**a**) Volcano plot of differentially expressed genes (DEGs) in normal and albino seedlings. (**b**) Heatmap of DEGs between normal and albino seedlings. NS: normal seedlings; AS: albino seedlings. The red and green dots correspond to upregulated and downregulated genes, respectively (**a**).

The alignment efficiency of clean reads with the Chinese plum reference genome varied from 87.69% to 95.2% using HISAT2. Based on the mapped reads, a total of 4048 DEGs (adjusted *p*-value < 0.05, |log_2_foldchange| > 1) were identified between normal and albino leaves, including 1987 upregulated and 2061 downregulated DEGs (Figure 5a). As shown in Figure 5b, genes with similar expression patterns via heatmap analysis were clustered into the same category among these DEGs in the normal and albino seedling leaves.

The functions of these DEGs were classified according to the GO database. Gene ontology (GO) analysis revealed that the upregulated DEGs were enriched in the category of molecular function (MF), which contained 13 GO terms, such as “UDP-glycosyltransferase activity, polygalacturonase activity” and “active transmembrane transporter activity”. For the 2061 downregulated DEGs, GO analysis revealed that they were enriched in two categories: molecular function (MF) and biological process (BP). The downregulated DEGs enriched in MF included “heme binding”, “tetrapyrrole binding”, “FMN binding”, “iron ion binding”, “oxidoreductase activity”, and 11 other GO terms. The downregulated DEGs in BP contained six GO terms, such as “glutathione metabolic process”, “cellular modified amino acid metabolic process”, and “ion transport” (Figure S1).

In addition, all DEGs were matched and assigned to 116 KEGG pathways. In albino seedlings, the downregulated DEGs were significantly enriched in pathways including “Flavonoid biosynthesis”, “Glutathione metabolism”, “Photosynthesis-antenna protein”, “Cysteine and methionine metabolism”, “Amino sugar and nucleotide sugar metabolism”, and “Plant circadian rhythm”, while no pathways were found to be significantly enriched for the upregulated DEGs (Figure S2).

### 2.7. Expression of Key Genes Involved in Photosynthesis

Photosynthesis is essential for the survival and development of plantlets. For the nuclear genes involved in the photosynthesis pathway, seven genes, including *psbP*, *psbQ-1*, *psbS*, *psb27*, *psb28*, *petF*, and *atpG*, were significantly upregulated, while three genes, including *psaK*, *psbQ-2*, and *atpD*, were significantly downregulated in the albino seedlings, according to the results of the transcriptome analysis. Moreover, seven DEGs belonging to light-harvesting chlorophyll a/b binding protein were identified as being involved in the pathway of photosynthetic antenna proteins; almost all of them were significantly downregulated, and only one gene was upregulated (Table S2).

**Figure 6:**
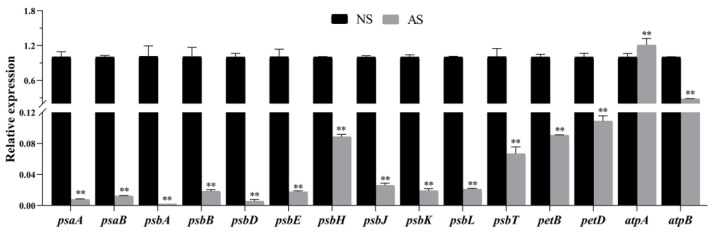
Expression of key genes associated with photosynthesis in leaves of normal and albino seedlings of *P. salicina* by qRT-PCR. The relative expression level of each gene was normalized using a combination of *EF1α* and *GAPDH* genes as an internal control. NS: normal seedlings; AS: albino seedlings. Error bars represent mean ± SD (*n* = 3). Asterisks indicate the following: ** *p <* 0.01.

To further reveal the accurate expression pattern of the photosynthesis-related chloroplast genes in the albino and normal seedlings, several chloroplast genes involving photosystem I/II, the cytochrome b/f complex, and ATP synthase were selected as representatives to perform the qRT-PCR analysis. The results showed that all the selected photosystem I- and II-related chloroplast genes (*psaA, psaB, psbA, psbB, psbD, psbE, psbH, psbJ, psbK, psbL,* and *psbT*) were significantly downregulated in the albino seedlings. Additionally, the ATP synthase-related gene exhibited contrary expression patterns; the *atpA* gene increased and *atpB* gene decreased in the albino seedlings. Furthermore, two genes related to the cytochrome b/f complex, *petB* and *petD*, were both significantly decreased. These findings indicate distinct expression patterns of photosynthesis-related genes in albino seedlings, suggesting that their photosynthetic processes are impaired (Figure 6).

### 2.8. Identification of DEGs Related to Chloroplast Development and Chlorophyll Biosynthesis

Chloroplast development and division have a close relationship with the albino plant. The DEGs regulating chloroplast development and division were identified according to the transcriptomic data. The *GLK1* gene that regulates chloroplast development and five *Ftsz* genes which are involved in chloroplast division were all significantly decreased in the albino seedlings (Table S2).

Chlorophylls are essential to photosynthesis, light harvesting, and energy transduction. In this study, five DEGs related to chlorophyll biosynthesis and degradation were identified. Among them, *SGRL* and *NOL* genes, which were closely related to chlorophyll degradation, were significantly upregulated in the albino seedlings. Similarly, three DEGs, including *HEME*, *POR*, and *UPM* genes, which were associated with chlorophyll biosynthesis, also exhibited higher expression levels in albino seedlings (Table S2).

### 2.9. Expressions of Key Genes Involved in Pigment Biosynthesis in Leaves of Seedlings

**Figure 7:**
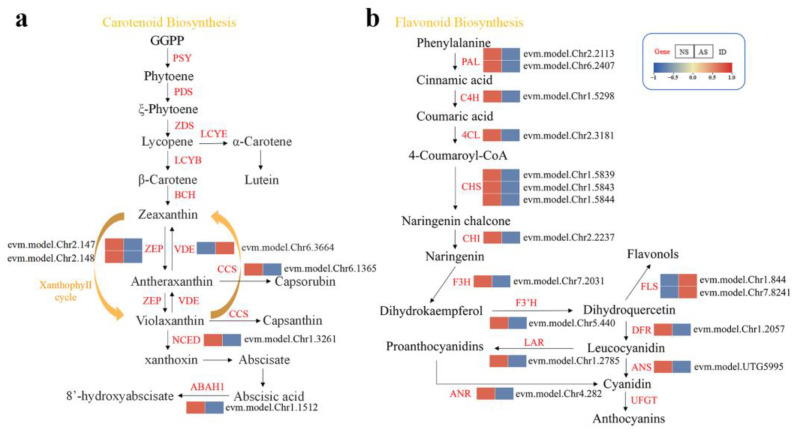
Expression profiles of DEGs associated with pigment biosynthesis in the leaves of normal and albino seedlings. (**a**) Expression profiles of DEGs involved in carotenoid biosynthesis. (**b**) Expression profiles of DEGs involved in flavonoid biosynthesis.

The expression of genes involved in carotenoid biosynthesis was analyzed, and six DEGs were identified. It was noteworthy that DEGs which were involved in the module of the ABA biosynthesis were all downregulated in the albino seedlings, except the *VDE* gene. As the key genes of the xanthophyll cycle, the expression levels of the *VDE* and *ZEP* genes showed contradictory change trends in the albino seedlings (Figure 7a). Moreover, two DEGs that were involved in ABA degradation and Capsanthin/Capsorubin synthesis both exhibited significantly decreased expression in albino seedlings. A total of 16 DEGs associated with flavonoid biosynthesis were identified; fourteen of them exhibited reduced expression levels, while the other two *FLS* genes were significantly upregulated in the albino seedlings (Figure 7b).

### 2.10. Validation of qRT-PCR

**Figure 8:**
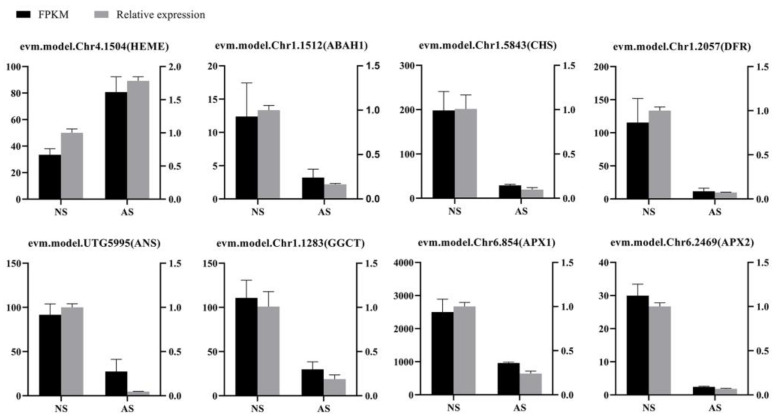
qRT-PCR validation of DEGs identified by RNA-seq in leaves of normal and albino seedlings of *P. salicina*. Black histograms represent RNA-seq data; gray histograms are qRT-PCR data. NS: normal seedlings; AS: albino seedlings. Error bars represent mean ± SD (*n* = 3).

To further validate the reliability of the transcriptome data, the DEGs involved in the metabolic pathways associated with albinism were selected to investigate the expression patterns by performing qRT-PCR analysis (Table S2). The expression of eight DEGs was detected, including a *HEME* gene involved in chlorophyll biosynthesis (evm.model.Chr4.1504), a *ABAH1* gene involved in carotenoid biosynthesis (evm.model.Chr1.1512), three genes involved in flavonoid biosynthesis, *CHS* (evm.model.Chr1.5843), *DFR* (evm.model.Chr1.2057), and *ANS* (evm.model.UTG5995), one *CCGT* gene involved in glutathione metabolism (evm.model.Chr1.1283), and two *APX* genes involved in ascorbate and aldarate metabolism (evm.model.Chr6.854, evm.model.Chr6.2469). The relative expression levels of these genes were consistent with the transcriptome sequencing results, confirming the reliability of the transcriptome data (Figure 8).

## 3. Discussion

In this study, the albino phenomenon during the seed propagation of *P. salicina* was systematically investigated; the comparative analyses of morphology, cytology, photosynthetic physiology, and transcriptomics between albino and normal seedlings provide new insights into the albino mechanism of *P. salicina.*

This research revealed significant variations in the proportion of albino seedlings among different varieties of *P. salicina,* which might be correlated with the potential genetic differences among varieties. Previous studies in other perennial plant species found that albinism was a qualitative character and controlled by one or more recessive alleles [28,29,30,31], and a similar genetic mechanism probably also occurred in *P. salicina*. According to the proportion of albino seedlings in the total germination, the varieties of *P. salicina* in this research could be divided into three distinct types with a high albino rate (>25%, e.g.,‘Fengtangli’), low albino rate (e.g.,‘Sanhuali’), and no albino (e.g.,‘Sanyueli’). Similar differences in the proportions of the albino seedlings were also observed among distinct varieties of plantain [30] and sweet cherry [31]. The varieties with a high albino rate were not ideal selections as the parent in hybridization breeding, since the progeny population size had to be significantly enlarged and the breeding efficiency was affected. However, constructing several hybridization populations between *P. salicina* varieties with a high albino rate and varieties with no albino was helpful to further reveal the genetic factors involved in the differences in the albino rate.

Significantly lower contents of chlorophyll and carotenoid were detected in leaves of albino seedlings. Compared to Chlb, Chla exhibited a more significant decrease in albino seedlings, and the Chla/Chlb ratio was also significantly reduced accordingly, which might be due to a faster hydrolysis ratio of Chla compared with Chlb in albino seedlings. The Chla/Chlb ratio, which is related to antenna size, could be an indicator of functional pigment equipment [32]. The significant decline of the Chla/Chlb ratio was also widely found in albino tissues of different species [23,33], which could be correlated with the abnormal structures of the photosynthetic apparatus.

These photosynthetic pigments are crucial for converting light energy into chemical energy during photosynthesis. Since photosynthetic pigments are primarily present in the chloroplasts of plant cells, their absence may be accompanied by a series of changes in the chloroplast. Similar to previous studies on albino leaves, the albino seedlings produced from *P. salicina* seeds showed a significant reduction in the number of chloroplasts compared to normal seedlings. In normal seedlings, several chloroplasts were present in each cell, whereas albino seedlings had only a small number of chloroplasts. Additionally, the chloroplasts in albino seedlings exhibited abnormal features, including more vesicles, fragmented lamellar structures, and numerous osmiophilic granules. These observations are consistent with previous studies on albino seedlings. Thylakoids play a pivotal role in photosynthesis, particularly in the absorption and conversion of light energy [34]. Previous studies have shown that chloroplasts in albino plants often exhibit abnormal thylakoid structures [35] and, in some studies, can hardly be defined [36]. The occurrence of vesicles and an increase in osmiophilic granules have also been observed in certain albino leaf chloroplasts. For example, in the chloroplast ultrastructure of *Ginkgo biloba* gold-colored mutant leaves, irregular vesicles and plentiful plastoglobules were similarly detected [37]. Differing levels of vesicles were identified in the chloroplasts of chlorophyll-deficient leaves in *Anthurium andraeanum* [38]. Similarly, in the albino seedlings of this study, the chloroplast vesicles also lacked significant additional structures. These structural abnormalities observed in chloroplasts prevent them from functioning properly in photosynthesis.

In this study, chlorophyll fluorescence parameters, including F_0_, Fm, FV/Fm, NPQ, and qP, which are important indicators of plant photosynthetic efficiency, were significantly reduced in albino seedlings. Among them, FV/Fm represents the maximum efficiency of PSII photochemistry and serves as an important indicator of photoinhibition. Similar to the results of this study, significant decreases in FV/Fm were also observed in *Arabidopsis* albino mutants [39] and transgenic albino poplar seedlings [13], indicating the different degrees of damage to the PSII reaction centers. NPQ represents the ability to dissipate excess light energy as heat during photosynthesis and serves as an indicator of non-photochemical quenching. F_0_ and Fm represent the baseline and maximum fluorescence signals under light intensity, respectively, while qP is an indicator of the electron transfer efficiency in photosynthesis [40]. The decreases in these parameters also reflect reduced light absorption, decreased photosynthetic efficiency, weakened electron transfer efficiency, and abnormal photosynthesis in albino seedlings. In grapevine leaves with iron-deficiency-induced chlorosis, F_0_ and FV/Fm were significantly lower compared to normal leaves [41]. Rice mutants with chlorophyll deficiency also showed a decreasing trend in FV/Fm, qP, and F_0_ [42]. Peach leaves with iron-deficiency-induced chlorosis exhibited decreases in FV/Fm and NPQ but an increase in qP [43]. Additionally, the net photosynthetic rate can be directly used to compare the photosynthetic capacity of plants. In this study, albino seedlings exhibited a significant reduction in the net photosynthetic rate, implying weakened photosynthetic ability. Similar decreases in Pn values were also observed in research on the albino phenotype in apple and arecanut [6,11].

Stomata play a crucial role in gas exchange, water regulation, temperature modulation, and signal transduction during photosynthesis and respiration in plants. Previous studies have shown differences in stomatal morphology, aperture, and density between chlorophyll-deficient leaves and normal leaves. For example, the leaves of chlorotic plants have smaller stomatal apertures compared to normal leaves [44], and the proportion of open stomata in albino rice is significantly lower than that in wild type [45]. Albino seedlings of royal poinciana exhibit poor differentiation of stomatal guard cells [8]. In our study, albino leaves exhibited relatively smaller stomatal apertures, and the guard cells of stomata appeared collapsed and poorly differentiated. There were also differences in stomatal density between albino leaves and normal leaves, although the specific patterns varied among different albino plants. In our study, the albino seedlings had lower stomatal density, consistent with studies on Agave [15], while albino leaves of royal poinciana had higher stomatal density [8]. The collapse of guard cells in albino seedlings may result in the inability to open and close stomata normally during photosynthesis, affecting the efficiency of photosynthesis. In addition, in this study, the stomatal conductance (Gs), intercellular CO_2_ concentration (Ci), and transpiration rate (E) values in albino seedlings significantly increased compared to those in normal seedlings. This also implies that the stomata of albino seedlings might not function normally in regulating the movement of gases in and out. Similar results were also found in an albino mutant of *Agave angustifolia* [15] and albino propagules in *Kandelia obovata* [23].

The impaired photosynthetic activity in albino seedlings is often accompanied by changes in the biochemical substance content. In this study, the enzyme activities of CAT and POD were significantly higher in albino seedlings compared to normal seedlings, while SOD activity was decreased. SOD is the first line of defense in plants for scavenging reactive oxygen species and can convert oxygen radicals into hydrogen peroxide (H_2_O_2_), while CAT and POD mainly scavenge H_2_O_2_ and peroxides. In some iron-deficient chlorotic plants, CAT and POD activity often decrease while SOD activity increases, such as in sunflower [46] and peanut [47]. The enzyme activity in the albino seedlings 15 days after sowing indicates that POD and CAT play a more important role in removing hydrogen peroxide and peroxides accumulated in albino seedlings. Significantly increased levels of oxidative stress were also found in the albino tissues of various plant species [23,48,49,50]. The photooxidative protection was reduced in albino seedlings, and their photosynthetic machinery was easily affected; the physiological condition of albinism can induce different stressful conditions in plants, with a resultant outburst in the production of ROS [48]. Therefore, we inferred that the excessive ROS accumulated in albino seedlings triggered the antioxidant system in response to oxidative stress. The accumulation of these substances may lead to the browning and withering of leaves and ultimately results in death. PRO and ASA may also be involved in the antioxidative process of albino seedlings, thus exhibiting different behavior compared to normal seedlings.

Albino plants can be generated by impaired chloroplast assembly or deficiency in chlorophyll metabolism. Many studies have shown that albino plants usually have significant changes in the microstructure of chloroplasts, and the chloroplast differentiation and development were impaired. *GLK* and *Ftsz* are two key gene families which regulate chloroplast development and division [22,51]. It has been reported that *GLK* genes regulate chloroplast development in several plant species such as rice, maize, and *Arabidopsis* [52]. Moreover, many studies found that the expression levels of *GLK* genes in albino mutants were lower than that in the wild type [21,22]. In the present study, the expression level of the *GLK* gene was significantly decreased in albino seedlings compared with normal seedlings, similar to reports of other species. The *Ftsz* gene family is essential for chloroplast division in higher plants [53,54]. In this study, five *Ftsz* genes exhibited significantly reduced expression levels in the albino seedlings. Therefore, inhibition of the *GLK* and *Ftsz* genes might be partly responsible for the impaired chloroplast development and division observed in the albino seedlings in our study.

*NOL* and *SGR* genes are essential for the degradation of chlorophyll; their corresponding gene defective mutants exhibited a stay-green phenotype, and the chlorophyll degradation was affected during senescence [55,56]. In this study, the higher expression of these two genes in the albino seedlings suggested that the breakdown of chlorophyll was accelerated. For the genes involved in chlorophyll biosynthesis, many of them were downregulated in different reported albino mutants [22,57,58,59]. However, the chlorophyll-biosynthesis-related DEGs identified in this study, such as *HEME* and *POR*, all exhibited upregulated expression in the albino seedlings. Similarly, the higher expression levels of several chlorophyll-biosynthesis-related genes were also observed in the *alc* albino cucumber mutant [60], *mta* albino wheat mutant [21], and the complete white leaves of *Ananas comosus* var. *Bracteatus* [61]. The increased expression of these genes might be a response to the accelerated chlorophyll degradation in the albino tissues.

Photosynthesis in higher plants is associated with a series of functional protein complexes like the light-harvesting chlorophyll protein complex and the reaction center complexes of photosystem I and photosystem II, which requires the coordination of chloroplast genes and nuclear genes [51,58]. For the nuclear genes, the DEGs encoding photosynthetic antenna proteins had significantly reduced expression levels in the albino seedlings, which might partly account for the reduced photosynthetic capacity of the albino seedlings. For the chloroplast genes, the chloroplast has its own transcription systems, and the chloroplast genes can be divided into PEP (plastid-encoded RNA polymerase)-dependent genes and NEP (nuclear-encoded plastid RNA polymerase)-dependent genes, according to the types of RNA polymerases [62]. Interestingly, the chloroplast genes that are involved in photosystem I and II, which were classified into the PEP-dependent genes [63], were significantly decreased in the albino seedlings in the qRT-PCR analysis. The selected chloroplast genes that are involved in the cytochrome b/f complex and ATP synthase, which belong to other types, exhibited distinct expression patterns in albino seedlings.

The PEP-dependent gene mainly participates in photosynthesis at the later stages of chloroplast development [63]. The function of the PEP complex was strongly associated with chloroplast development and plant growth [64]. The inactivation of genes that encode the accessory proteins in the PEP complex could lead to an albino phenotype and impaired chloroplast development, and the abundance of PEP-dependent plastid transcripts is reduced in the deficient lines of these genes. For example, the expression of PEP-dependent genes was strongly affected in rice *osppr16* and *Arabidopsis ptac10* and *tac7-2* mutant plants which exhibited the albino phenotype [63,65,66]. In this study, the significant decline in the expression levels for the examined PEP-dependent chloroplast genes in the albino seedlings might be also due to the potential gene mutations that give rise to the dysfunction of the PEP complex.

Carotenoids act as antenna pigments in photosynthesis and have the function of light protection and free radical scavenging [67]. In this present study, six DEGs associated with carotenoid biosynthesis were identified, and five of them were downregulated; the change in the carotenoid metabolic pathway might be a positive adaptation to the adverse effects caused by chlorophyll deficiency or abnormal chloroplast. Carotenoids are also the synthetic precursors of plant hormones such as abscisic acid. The downregulation of the ABA-biosynthesis-related DEGs might lead to a reduction in ABA levels. Owing to the inhibition of ABA synthesis, the accumulated ROS might not be removed in time and then result in damage to the developing chloroplast structure [68]. Meanwhile, the *ABAH1* (abscisic acid 8′-hydroxylase 1) gene encoding the enzyme that catalyzes the pivotal step in the oxidative degradation of ABA [69] also had significantly reduced expression levels in the albino seedlings in this study. Several previous studies indicated that appropriate ABA content could rescue albino mutants [6,70]. Therefore, the downregulation of the ABA-degradation-related *ABAH1* gene might be a response to the albino which aimed to maintain the balance of the ABA content. PRO and ASA may also be involved in the antioxidative process of albino seedlings, thus exhibiting different behavior compared to normal seedlings.

The xanthophyll cycle is an important mechanism that protects photosynthetic tissues from photodamage through the dissipation of excess light energy. Two main reactions catalyzed by the enzyme of *VDE* and *ZEP* are included in the xanthophyll cycle. In this study, the expression of the two *ZEP* genes were significantly downregulated, while that of the *VDE* gene was upregulated in albino seedlings, and the equilibrium of the xanthophyll cycle might be significantly affected in albino seedlings. Similar expression patterns were also observed in the bleached parts of the striped leaves of rice leaf color mutant *B03S* [68]. The significant changes in expression levels of the key biosynthesis genes were usually accompanied by the excessive accumulation of zeaxanthin, which might be an adaptive protective mechanism to enhance the thermal dissipation capacity and alleviate the adverse effects of albino seedlings. It has been reported that the xanthophyll cycle is assumed to be involved in the NPQ of excess light energy in the antenna of PS II, and the antenna proteins are an essential modulator of the xanthophyll cycle [71]. Therefore, the significant downregulation of the series of genes that encode antenna proteins might be associated with a disorder of the xanthophyll cycle and the decrement of NPQ value in albino seedlings.

Flavonoids, such as anthocyanins, flavonols, and proanthocyanidins, are also involved in regulating the color formation of various organs of plants. In this study, most of the DEGs that were involved in flavonoid biosynthesis had reduced expression levels in albino seedlings, indicating that the flavonoid biosynthesis was inhibited, which might be another factor that affects the color of the seedlings. Similarly, the inhibition of the genes involved in flavonoid biosynthesis was also observed in albinism tissues of other plant species like arecanut and tea [6,72]. Interestingly, two *FLS* genes were significantly upregulated in albino seedlings, which might lead to the accumulation of flavonols. In the chlorophyll-deficient albino tea mutant ‘Baiye 1′, flavonols were highly accumulated [72]. It has been widely reported that flavonols usually accumulate under oxidative stress and their biosynthesis might be driven by antioxidation under unfavorable circumstances [73]. Therefore, the upregulation of *FLS* genes in this study might be a response to the oxidative stress in albino seedlings.

## 4. Materials and Methods

### 4.1. Plant Material

This study collected seeds of different varieties of Chinese plum from Guiyang City, Guizhou Province (‘Fengtangli’, ‘Fenghuangli’, ‘Wushancuili’, and ‘Ziyeli’), and Guangzhou City, Guangdong Province (‘Sanhuali’ and ‘Sanyueli’). All the above-mentioned seeds were produced through natural pollination. Stones were carefully cracked using a hammer to obtain the plum embryos. Intact embryos were selected and soaked in water until they absorbed enough moisture. The plump embryos were wrapped in moist paper towels and placed in covered culture dishes. The dishes were kept in a dark environment at a low temperature of 4 °C. Every 3 days, the status of the embryos was observed, and any moldy seeds were promptly removed. The growth of the root of the embryo was awaited, which can take several months. During this period, the moist paper towels needed to be replaced every week. When the roots of the embryos grew to approximately 0.5 cm in length, they were transplanted into pots (6.5 cm × 6.5 cm) filled with sterile substratum containing peat, sand, perlite, and vermiculite (3:1:1:1). After germination, the seedlings were used for the further comprehensive observations and measurements. The cultivation environment was in a greenhouse with a temperature of 26 °C, a photoperiod of 16 h per day, and a light intensity of 3000 lx.

### 4.2. Seed Germination Assessment and Morphological Data Measurement

Ten days after sowing, the proportions of normal seedlings and albino seedlings were recorded in the total germinated seedlings for each variety. Fifteen days after sowing, the length and width of the largest leaf, plant height, and stem thickness were measured for albino seedlings and normal seedlings.

### 4.3. Quantification of Chlorophyll and Carotenoid

Fifteen days after sowing, the fresh leaf samples from normal and albino seedlings (0.1 g) were collected and ground into powder in liquid nitrogen, with a mixed solution of 1 mL of acetone and water (80%) for 24 h under dark conditions, shaken for 30 s every 8 h. The supernatants were collected by centrifugation, and the concentrations of chlorophyll a, chlorophyll b, chlorophyll, and carotenoid at 663 nm, 646 nm, and 470 nm were determined with an ultra-micro spectrophotometer (Biotek Cytation5, Biotek, Winooski, VT, USA). The contents of chlorophyll a, chlorophyll b, chlorophyll, and carotenoid were calculated according to published protocols [74].

### 4.4. Transmission Electron Microscopy (TEM) Observation

The fresh leaf samples from normal and albino seedlings of prunus were cut into small pieces (1 mm × 1 mm), first fixed with 2.5% glutaraldehyde (GA) at 4 °C in the phosphate buffer (pH 7.2), washed in the phosphate buffer, and then fixed with 1% OsO_4_; these samples were dehydrated through a graded series of ethanol and acetone infiltration by Spurr resin. The samples were stained with 1% (*w*/*v*) aqueous uranyl acetate and lead citrate solutions and observed with a transmission electron microscope of Model Talos L120C (Thermo Fisher Scientific, Cleveland, OH, USA).

### 4.5. Measurement of Chlorophyll Fluorescence and Photosynthetic Parameters

Fifteen days after sowing, the seedlings were used to investigate the parameter of chlorophyll fluorescence with fluorescence imaging apparatus (PhenoVation B.V., Wageningen, The Netherlands). They were kept in the dark for 15 min before measuring the chlorophyll fluorescence parameters of both albino and normal seedlings (*n* = 3). The parameters measured included the initial fluorescence (F_0_), maximum fluorescence (Fm), non-photochemical quenching coefficient (NPQ), photochemical quenching coefficient (qP), and maximum photochemical efficiency of PSII (Fv/Fm). Data were obtained in triplicate from the interval region of the entire plant between 8:30 and 10:00 a.m.

Fifteen days after sowing, we selected the largest leaves from normal and albino seedlings to determine photosynthetic parameters between 9 and 11 a.m. These photosynthetic parameters, including the net photosynthesis rate (Pn), stomatal conductance (Gs), intercellular CO_2_ concentration (Ci), and transpiration rate (E), were measured using a portable photosynthesis system (CIRAS-3, PP-System, Amesbury, MA, USA). These photosynthetic parameters were recorded when the rate of CO_2_ uptake had stabilized.

### 4.6. Scanning Electron Microscopy (SEM) Observation

Fresh leaf samples were obtained from 15-day-old normal and albino seedlings. These samples were quickly cut into small pieces (3 mm × 3 mm) using a sharp blade within 1–3 min. Subsequently, they were then fixed with an electron microscopy fixative (Servicebio, Wuhan, China) and stored at 4 °C for preservation. The samples were then dehydrated through a graded series of ethanol (30%, 50%, 70%, 80%, 90%, and 100%) and subjected to critical point drying. Afterward, these samples were attached to metallic stubs using carbon stickers and sputter-coated with gold for 30 s, then observed with scanning electron microscopy.

### 4.7. Determination of Biochemical Substances Content and Enzyme Activity

The activities of POD, CAT, and SOD, as well as the content of GSH, ASA, and PRO, were determined using commercially available assay kits (Comin Biotechnology, Suzhou, China). Fresh leaf samples from normal and albino seedlings (0.1 g) were collected, ground under liquid nitrogen, and 1 mL of extract was added. The mixture was then homogenized in an ice bath and centrifuged at 8000× *g* at 4 °C for 10 min. The supernatant was collected and kept on ice for testing. Subsequent experimental procedures followed the instructions provided in the kit (www.cominbio.com, accessed on 20 May 2023). After preheating the microplate reader (Cytation 5, BioTek, Winooski, VT, USA) for more than 30 min, the wavelength was adjusted to the specific wavelength for each enzyme and biochemical substance (POD: 470 nm; CAT: 405 nm; SOD: 450 nm; GSH: 412 nm; ASA: 420 nm; PRO: 520 nm) before completing the assessment.

### 4.8. Transcriptome Sequencing and Analysis

Leaf samples of 15-day-old seedlings were selected for transcriptome sequencing. RNA was extracted using the QIAGEN TianGen Polyphenol and Polysaccharide Kit (Qiagen, Hilden, Germany). Three biological replicates were established for each sample. RNA quality control was performed using the Agilent 2100 Bioanalyzer (Agilent Technologies, Santa Clara, CA, USA). After library construction and quality assessment, sequencing was carried out using the Illumina NovaSeq 6000 platform (Illumina, San Diego, CA, USA). The reference genome data for ‘Sanyueli’ was used as a reference (https://www.rosaceae.org/Analysis/9450778, accessed on 23 May 2023) [75]. Alignment was performed using HISAT2 v2.0.5. Novel transcripts were predicted using StringTie (version 1.3.3b) [76], and FPKM values were calculated to estimate gene expression levels. Differential expression analysis was conducted using the DESeq2 software (version 1.20.0), with genes having an adjusted *p*-value < 0.05 considered as differentially expressed. The differentially expressed genes were subjected to GO enrichment analysis and KEGG pathway enrichment analysis using the clusterProfiler software (version 3.8.1).

### 4.9. Validation through Fluorescence qRT-PCR Analysis

Quantitative Reverse Transcription PCR (qRT-PCR) experiments were used to confirm and analyze the basic expression levels of a subset of candidate genes. The RNA was reverse-transcribed and synthesized into cDNA using the Hifair^®^ III 1st Strand cDNA Synthesis SuperMix for qPCR (gDNA digester plus) kit. The qRT-PCR was carried out with the Roche Lightcyler^®^ 480 instrument using SYBR Green Master Mix (Vazyme, Jiangsu, China). Primers were designed for qRT-PCR using Primer Express 3.0 software (Applied Biosystems, Waltham, MA, USA), and the primer sequences are shown in Table S3. A combination of *EF1α* and *GAPDH* genes, which were identified as the best reference genes for transcript normalization in vegetative tissues of Chinese plum [77], was selected as the internal control in this research. The reaction was carried out as follows: 95 °C for 30 s, followed by 40 cycles of 95 °C/10 s, and 60 °C/30 s. Each reaction was performed in three biological replications, and the relative gene expression values were calculated using the 2^−△△CT^ method.

### 4.10. Statistical Analysis

All data were obtained from three independent biological replicates for each sample, with each biological replicate having three technical replicates. The data were analyzed using the Student’s *t*-test in IBM SPSS Statistics 26 (IBM Corp., Armonk, NY, USA). Data are shown as mean ± SD.

## 5. Conclusions

In this study, the morphological, cytological, and physiological characteristics and gene expression profiling were comparatively analyzed in normal and albino seedlings of *P. salicina*. The albino seedlings could not grow normally and exhibited lethal characteristics; differences in the proportions of albinos were observed among different plum varieties. Abnormal chloroplast ultrastructure and smaller and sparser stomata with abnormal morphology were observed in the albino seedlings. The chlorophyll and carotenoid content, the chlorophyll fluorescence parameters, and the net photosynthetic rate were significantly reduced, indicating that the photosynthetic capacity was impaired and photosynthesis was hindered in albino seedlings. Additionally, the stomatal conductance, intercellular CO_2_ concentration, and transpiration rate increased significantly, also corroborating the abnormal stomatal structure in albino seedlings. Moreover, the changes in antioxidant enzyme activities and content of proline and antioxidants suggested physiological disturbances in albino seedlings. The transcriptome and qRT-PCR analyses revealed the unique characteristics of the albinism mechanism in *P. salicina*. For most of the genes related to chlorophyll and carotenoid biosynthesis, no significant differences in gene expression were found, while a series of genes associated with flavonoid biosynthesis were significantly downregulated in albino seedlings. The downregulation of *GLK* and *Ftsz* genes might be responsible for the impaired chloroplast development and division in the albino seedlings. The significant downregulation of the nuclear genes encoding photosynthetic antenna proteins and the PEP-dependent chloroplast genes involved in photosystem I and II could give rise to the notably reduced photosynthetic capacity of the albino seedlings. Our findings provide insights into the molecular mechanism underlying the albino phenotype in *P. salicina*, which will facilitate the improvement of reproductive breeding efforts.

## Figures and Tables

**Table 1:** Chlorophyll fluorescence, photosynthetic parameters, and transpiration rate in leaves of seedlings.

	Chlorophyll Fluorescence Parameters				
Phenotype	F_0_	Fm	Fv/Fm	NPQ	qP
NS	3352.33 ± 246.07	11170.67 ± 831.43	0.74 ± 0.01	0.58 ± 0.11	0.60 ± 0.01
AS	1618.00 ± 421.23 **	1749.00 ± 605.87 **	0.15 ± 0.04 **	0.25 ± 0.01 **	0.45 ± 0.17
	Photosynthetic parameters			E (mmol H_2_O m^−2^s^−1^)	
Phenotype	Pn (μmol CO_2_ m^−2^s^−1^)	Gs (mmol H_2_O m^−2^s^−1^)	Ci (μmol mol^−1^)		
NS	2.73 ± 0.15	90.67 ± 2.08	349.00 ± 2.00	2.11 ± 0.04	
AS	−2.50 ± 0.70 **	400.33 ± 35.73 **	447.67 ± 23.50 **	5.26 ± 0.25 **	

NS: normal seedlings; AS: albino seedlings; F_0_: the initial fluorescence; Fm: maximum fluorescence; Fv/Fm: maximum photochemical efficiency of PSII; NPQ: non-photochemical quenching coefficient; qP: photochemical quenching coefficient; Pn: net photosynthetic rate; Gs: stomatal conductance; Ci: intercellular CO_2_ concentration; E: transpiration rate. Error bars represent mean ± SD (*n* = 3). Asterisks indicate the following: ** *p* < 0.01.

## Data Availability

The raw reads from the Illumina Novaseq 6000 platform were deposited into NCBI in the Sequence Reads Archive (SRA) under the BioProject accession number PRJNA1005015.

## Supplementary Materials

The following supporting information can be downloaded at https://www.mdpi.com/article/10.3390/ijms241914457/s1.
