# Supplementary material for: Integrated Cytological, Physiological, and Transcriptome Analyses Provide Insight into the Albino Phenotype of Chinese Plum (Prunus salicina)
Source: Int J Mol Sci. 2023 Sep 22;24(19):14457. doi: 10.3390/ijms241914457 (PMC10573071; doi:10.3390/ijms241914457)
Supplement: Supplementary file 1 [file ijms-24-14457-s001.zip › Supplementary Figures.pdf]

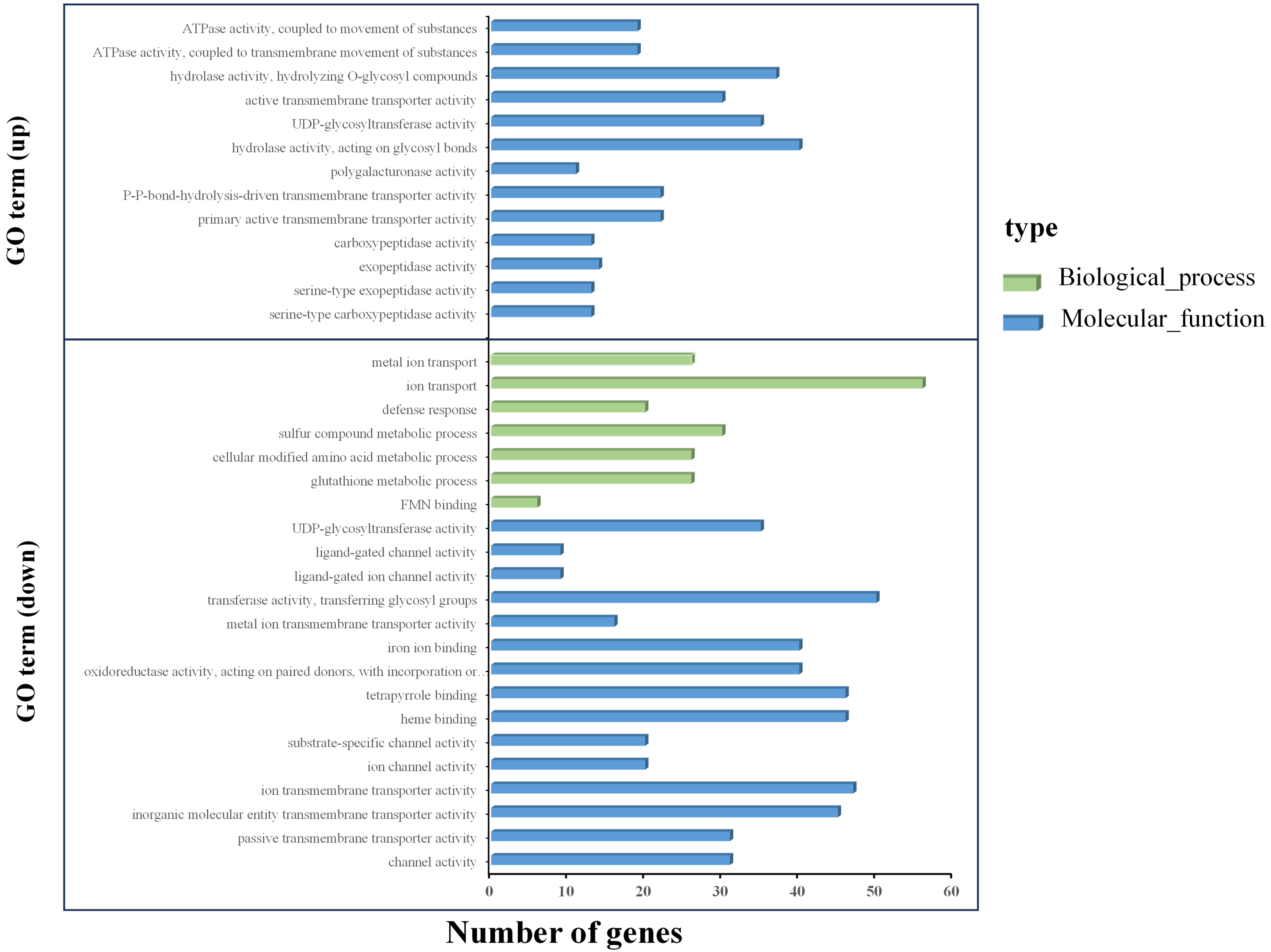

Figure S1. GO enrichment analysis of DEGs between normal and albino seedlings.

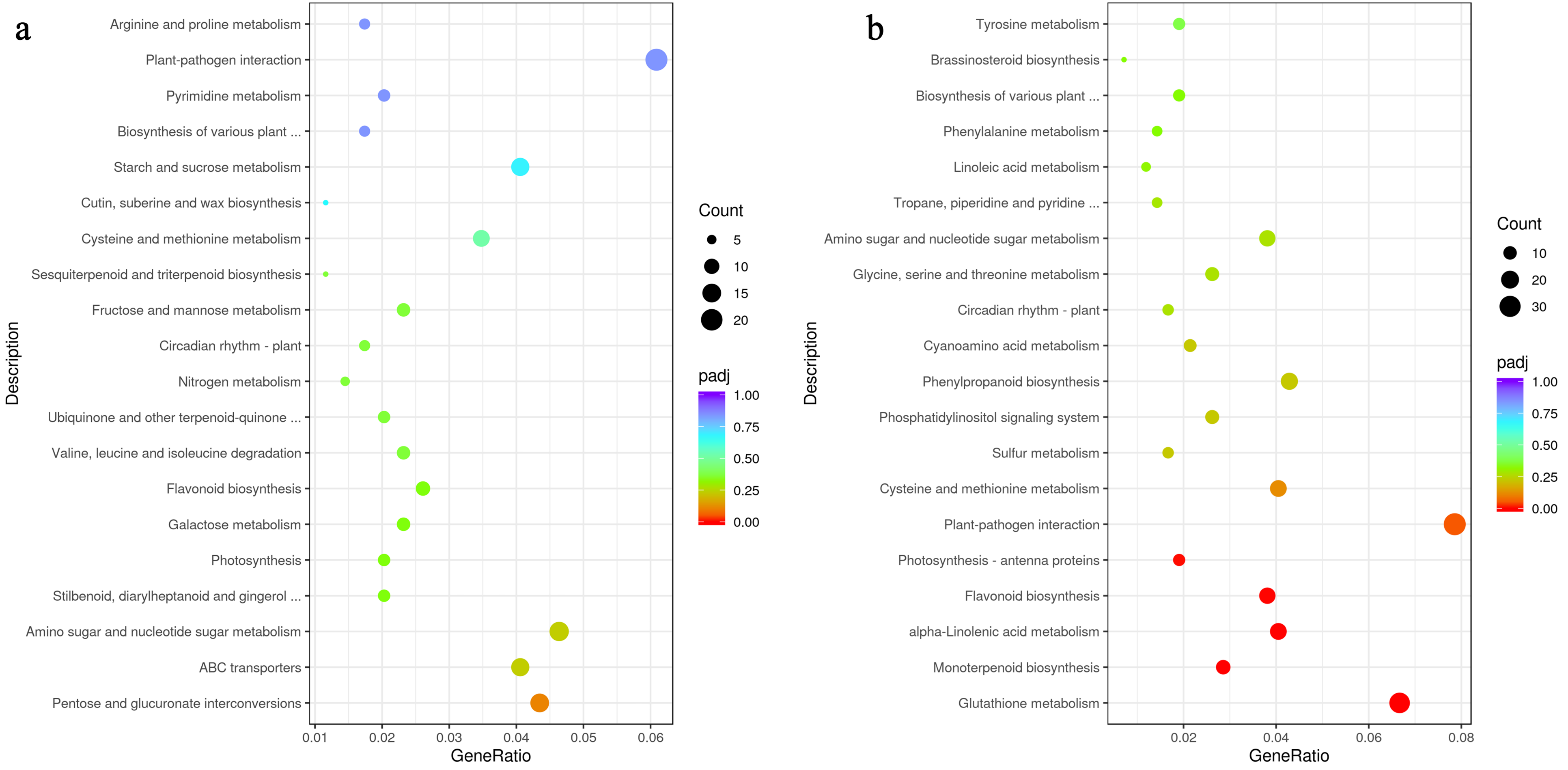

Figure S2. KEGG pathway enrichment analysis of DEGs between normal and albino seedlings. (**a**) The upreguated DEGs. (**b**) The downregulated DEGs.
