# Supplementary material for: Integrated Cytological, Physiological, and Transcriptome Analyses Provide Insight into the Albino Phenotype of Chinese Plum (Prunus salicina)
Source: Int J Mol Sci. 2023 Sep 22;24(19):14457. doi: 10.3390/ijms241914457 (PMC10573071; doi:10.3390/ijms241914457)
Supplement: Supplementary file 1 [file ijms-24-14457-s001.zip › Table S1.pdf]

**Table S1.** Summary of RNA-seq data for all samples (NS: normal seedlings; AS: albino seedlings)

| Sample name | AS1 | AS2 | AS3 | NS1 | NS2 | NS3 |
|---|---|---|---|---|---|---|
| Raw reads | 45814168 | 42688558 | 49928860 | 44625318 | 40903168 | 47002140 |
| Raw bases | 6.87G | 6.4G | 7.49G | 6.69G | 6.14G | 7.05G |
| Clean reads | 43502554 | 41516750 | 47879454 | 44577456 | 39749774 | 45336072 |
| Clean bases | 6.53G | 6.23G | 7.18G | 6.69G | 5.96G | 6.8G |
| Error rate(%) | 0.03 | 0.03 | 0.03 | 0.03 | 0.03 | 0.03 |
| Q20(%) | 97.13 | 97.33 | 96.99 | 97.46 | 97.33 | 97.27 |
| Q30(%) | 92.3 | 92.67 | 91.75 | 93.1 | 92.72 | 92.62 |
| GC content(%) | 45.76 | 45.6 | 45.66 | 48.21 | 45.55 | 45.4 |
