# Supplementary material for: Integrated Cytological, Physiological, and Transcriptome Analyses Provide Insight into the Albino Phenotype of Chinese Plum (Prunus salicina)
Source: Int J Mol Sci. 2023 Sep 22;24(19):14457. doi: 10.3390/ijms241914457 (PMC10573071; doi:10.3390/ijms241914457)
Supplement: Supplementary file 1 [file ijms-24-14457-s001.zip › Table S2.pdf]

Table S2. Genes with altered levels in albino seedlings as mentioned in this study

Function	Gene ID	Gene family	NS_FPKM	AS_FPKM	log_2FoldChange	padj	Expression level in albino seedling	Annotation
Flavonoid biosynthesis	evm.model.Chr7.8241	FLS	0.18	1.85	3.33	0.0302	Up-regulated	flavonol synthase
	evm.model.Chr1.844	FLS	4.43	18.77	2.07	1.73E-09	Up-regulated	flavonol synthase
	evm.model.Chr2.2113	PAL	39.95	16.58	-1.27	0.0028	Down-regulated	Phenylalanine ammonia-lyase
	evm.model.Chr6.2407	PAL	80.59	34.03	-1.24	9.83E-08	Down-regulated	Phenylalanine ammonia-lyase
	evm.model.Chr1.5298	C4H	1.91	0.01	-7.25	2.23E-06	Down-regulated	Trans-cinnamate 4-monooxygenase
	evm.model.Chr2.3181	4CL	41.13	7.71	-2.42	3.23E-18	Down-regulated	4-coumarate--CoA ligase
	evm.model.Chr1.5839	CHS	144.78	29.33	-2.30	3.38E-23	Down-regulated	Chalcone synthases 2
	evm.model.Chr1.5844	CHS	84.45	8.06	-3.39	1.69E-24	Down-regulated	Chalcone synthases 3
	evm.model.Chr1.5843	CHS	198.06	29.20	-2.76	2.72E-34	Down-regulated	Chalcone synthases 3
	evm.model.Chr2.2237	CHI	51.56	17.43	-1.57	3.71E-05	Down-regulated	Chalcone isomerase
	evm.model.Chr7.2031	F3H	169.38	29.65	-2.51	1.15E-16	Down-regulated	Flavanone 3-hydroxylase
	evm.model.Chr5.440	F3'H	407.42	168.29	-1.28	8.26E-10	Down-regulated	Flavonoid 3'-monooxygenase
	evm.model.Chr1.2057	DFR	115.38	11.50	-3.33	3.55E-18	Down-regulated	Dihydroflavonol 4-reductase
	evm.model.UTG5995	ANS	91.54	27.39	-1.74	0.0002	Down-regulated	Anthocyanidin synthase
	evm.model.Chr4.282	ANR	92.74	35.06	-1.40	6.22E-07	Down-regulated	Anthocyanidin reductase
	evm.model.Chr1.2785	LAR	39.99	12.36	-1.69	0.0027	Down-regulated	Leucanthocyanidin reductase
Chloroplast development	evm.model.Chr3.1291	GLK1	51.70	23.83	-1.12	0.0059	Down-regulated	Transcription activator GLK1

Chloroplast division	evm.model.Chr7.431	Ftsz	70.85	25.20	-1.49	1.16E-05	Down-regulated	Cell division protein FtsZ
	evm.model.Chr6.6061	Ftsz	280.05	109.31	-1.36	6.33E-06	Down-regulated	Cell division protein FtsZ
	novel.99	Ftsz	24.32	10.82	-1.17	0.0020	Down-regulated	Cell division protein FtsZ
	evm.model.Chr8.1626	Ftsz	320.26	70.82	-2.18	7.90E-15	Down-regulated	Cell division protein FtsZ
Carotenoid biosynthesis	evm.model.Chr2.147	ZEP	0.21	0.01	-3.90	0.1977	Down-regulated	Zeaxanthin epoxidase
	evm.model.Chr2.148	ZEP	2.00	0.03	-6.26	2.10E-06	Down-regulated	Zeaxanthin epoxidase
	evm.model.Chr6.3664	VDE	23.62	51.92	1.14	1.56E-06	Up-regulated	Violaxanthin de-epoxidase
	evm.model.Chr1.3261	NCED4	968.62	305.45	-1.66	3.86E-11	Down-regulated	9-cis-epoxycarotenoid dioxygenase 4
	evm.model.Chr1.1512	ABAH1	12.39	3.22	-1.95	4.50E-05	Down-regulated	Abscisic acid 8'-hydroxylase 1
	evm.model.Chr6.1365	CCS	41.64	15.81	-1.40	3.29E-05	Down-regulated	Capsanthin/capsorubin synthase
Chlorophyll biosynthesis	evm.model.Chr4.1504	HEME	33.44	80.69	1.27	1.80E-08	Up-regulated	Uroporphyrinogen decarboxylase
	evm.model.Chr4.1731	POR	157.14	542.94	1.79	2.27E-13	Up-regulated	Protochlorophyllide reductase
	evm.model.Chr2.1261	UPM	42.98	130.88	1.61	3.87E-08	Up-regulated	Uroporphyrin-III C-methyltransferase
Chlorophyll degradation	evm.model.Chr2.7872	NOL	16.17	36.67	1.18	1.19E-11	Up-regulated	Chlorophyll(ide) b reductase
	evm.model.Chr8.110	SGR	11.76	35.41	1.59	3.14E-13	Up-regulated	Protein Stay-Green
Photosynthesis	evm.model.Chr2.2265	psbP	25.25	77.74	1.62	1.69E-10	Up-regulated	Photosystem II oxygen-evolving enhancer protein 2
	evm.model.Chr7.2311	LHCA2	99.65	284.18	1.51	1.62E-15	Up-regulated	Light-harvesting complex I chlorophyll a/b binding protein 2

evm.model.Chr8.2229	psb28	86.44	241.66	1.48	1.14E-07	Up-regulated	Photosystem II 13kDa protein
evm.model.Chr3.2412	psb27	85.09	222.70	1.39	0.0246	Up-regulated	Photosystem II Psb27 protein
evm.model.Chr4.1861	psbQ-1	53.40	124.25	1.22	8.21E-06	Up-regulated	Photosystem II oxygen-evolving enhancer protein 3
evm.model.Chr4.1977	ATPF1G	5.84	13.18	1.18	2.73E-05	Up-regulated	F-type H+-transporting ATPase subunit gamma
evm.model.Chr5.2210	petF	54.83	117.04	1.09	9.41E-09	Up-regulated	Ferredoxin
evm.model.Chr1.904	psbS	300.10	623.78	1.06	2.74E-08	Up-regulated	Photosystem II 22kDa protein
evm.model.Chr4.1779	LHCB3	1459.52	645.39	-1.18	8.66E-13	Down-regulated	Light-harvesting complex II chlorophyll a/b binding protein 3
novel.202	LHCA3	2701.88	1122.83	-1.27	4.04E-17	Down-regulated	Light-harvesting complex I chlorophyll a/b binding protein 3
evm.model.Chr3.1865	LHCB2	10939.68	3994.22	-1.45	3.49E-05	Down-regulated	Light-harvesting complex II chlorophyll a/b binding protein 2
evm.model.Chr3.68	LHCB1	6556.21	2195.72	-1.58	0.0007	Down-regulated	Light-harvesting complex II chlorophyll a/b binding protein 1
evm.model.Chr3.681	LHCB1	6994.97	2197.87	-1.67	0.0012	Down-regulated	Light-harvesting complex II chlorophyll a/b binding protein 1
evm.model.Chr3.1866	LHCB1	1068.77	266.72	-2.00	2.02E-06	Down-regulated	Light-harvesting complex II chlorophyll a/b binding protein 1
evm.model.Chr4.828	psaK	3164.91	447.35	-2.82	1.08E-13	Down-regulated	Photosystem I subunit X
evm.model.Chr1.2395	psbQ-2	5.31	0.50	-3.36	2.76E-05	Down-regulated	Photosystem II oxygen-evolving enhancer protein 3
