# Supplementary material for: Integrated Cytological, Physiological, and Transcriptome Analyses Provide Insight into the Albino Phenotype of Chinese Plum (Prunus salicina)
Source: Int J Mol Sci. 2023 Sep 22;24(19):14457. doi: 10.3390/ijms241914457 (PMC10573071; doi:10.3390/ijms241914457)
Supplement: Supplementary file 1 [file ijms-24-14457-s001.zip › Table S3.pdf]

**Table S3.** Oligonucletide primer used in qRT-PCR assays in this study.

| Primer name | Forward sequence (5'-3') | Reverse sequence (5'-3') |
|---|---|---|
| QpsaA | CACAGGCATCCCAGGTAA | CGCCCGCTGAATAGAAAC |
| QpsaB | TATCGCTTATTCTGGTGT | ATTTCGGTTGTAGGTGTA |
| QpsbA | TGCCATTATTCCTACCTC | ACTAAGTTCCCACTCACG |
| QpsbB | TATGGACTAACGGGAAGG | TGGACGGACACTAAGATG |
| QpsbD | CCTATTTGGAAGGCTGTA | ACCTATTAGTCCGAAAGC |
| QpsbE | TTATTTATTGCGGGTTGG | CCTTGTCGGCTTTCTGTA |
| QpsbH | ACGAACTAATGCAGGGAG | ATAGAGCCATTGCGAGAC |
| QpsbJ | GGCCGATACTACTGGAAG | TACAGGGATGAACCTAAT |
| QpsbK | CCGCTCTTTATTCAAGTA | CTGGCATAACATCTACGA |
| QpsbL | CACAATCAAACCCGAACG | GGAAAATAAAACAGCAAGTA |
| QpsbM | CTCGCATTTATTGCTACT | ATCACCTTGGCTGACTGT |
| QpsbT | ATGGAAGCATTGGTTTAT | TTAGTTGGAACTTTAGGC |
| QPsatpA | GGAACGAACGGTTATCTT | GTCGGTAAATGTCTTGGTAG |
| QatpB | TATTTGGCGGAGTAGGTG | CTGGCGGTTCATTCATCT |
| QpetB | GGGTGTATTGACCGCATCT | AGCGTCTGGTACGCCTGT |
| QpetD | TCGTACAGTGCCAAATAA | AGGTAGTGTTGCTCCAAT |
| QPsHEME(evm.model.Chr4.1504) | TGCCATACAGCACCACAT | TTCGACCAGCTATCCCAC |
| QPsABAH1(evm.model.Chr1.1512) | AGCCGAATACATTTATACCA | ATCCCACTACTTCCCATC |
| QPsCHS(evm.model.Chr1.5843) | GGAAGTTCGCAAGGCTCA | ACGGAAGTAGTAGTCAGGGTA |
| QPsDFR(evm.model.Chr1.2057) | ATCCCAACTCTTGTGATTG | AGAGGTCGTCCAAGTGAA |
| QPsANS(evm.model.UTG5995) | AGCGTGACTTGTCCATTT | CTTCTCCAGCCTCCCTTC |
| QPsGGCT(evm.model.Chr1.1283) | CGATTGGAGAAGGCTATG | TGAGTGGGAGGTGGGTTG |
| QPsAPX1(evm.model.Chr6.854) | ATTCGGAACTATGAAGCACG | CTCAACCGCAACAACACC |
| QPsAPX2(evm.model.Chr6.2469) | CCTGGACAACCAACCCTC | TTCAGCATAATCCGCAAA |
| QPsEF1α | ATTGACAGGAGGTCTGGTAAGG | ATGGAGGGTACTCAGAGAAAGTC |
| QPsGAPDH | CGTAGCATCCACTCTCTAAATCC | TTCAGAGCTCCAAACCATAACA |
